# Resolution of severe gastroparesis induced by parasympathetic surge following facial trauma: a case report

**DOI:** 10.1186/s13256-024-04558-4

**Published:** 2024-05-16

**Authors:** Muhammad Haseeb-Ul-Rasool, Ahmed Elhawary, Utsow Saha, Arshia Sethi, Gowri Swaminathan, Hazem Abosheaishaa

**Affiliations:** Icahn School of Medicine at Mount Sinai, NYC Health+Hospitals Queens, New York, USA

**Keywords:** Gastroparesis, Intestinal motility disorder, Gastric emptying, Parasympathetic surge, A case report

## Abstract

**Background:**

Gastroparesis is a condition that affects the motility of the gastrointestinal (GI) tract, causing a delay in the emptying process and leading to nausea, vomiting, bloating, and upper abdominal pain. Motility treatment along with symptom management can be done using antiemetics or prokinetics. This study highlights the diagnostic and therapeutic challenges of gastroparesis and suggests a potential link between facial trauma and symptom remission, indicating the need for further investigation.

**Case presentation:**

A 46-year-old Hispanic man with hypertension, type 2 diabetes (T2D), and hyperlipidemia on amlodipine 10 mg, lisinopril 5 mg, empagliflozin 25 mg, and insulin glargine presented with a diabetic foot ulcer with probable osteomyelitis. During hospitalization, the patient developed severe nausea and vomiting. The gastroenterology team advised continuing antiemetic medicine and trying very small sips of clear liquids. However, the patient didn’t improve. Therefore, the gastroenterology team was contacted again. They advised having stomach emptying tests to rule out gastroparesis as the source of emesis. In addition, they recommended continuing metoclopramide, and starting erythromycin due to inadequate improvement**.** Studies found a 748-min stomach emptying time. Normal is 45–90 min. An uneventful upper GI scope was done. Severe gastroparesis was verified, and the gastroenterology team advised a percutaneous jejunostomy or gastric pacemaker for gastroparesis. Unfortunately, the patient suffered a mechanical fall resulting in facial trauma. After the fall, the patient’s nausea eased, and emesis stopped. He passed an oral liquids trial after discontinuation of erythromycin and metoclopramide.

**Conclusion:**

This case exemplifies the difficulties in diagnosing and treating gastroparesis. An interesting correlation between parasympathetic surges and recovery in gastroparesis may be suggested by the surprising remission of symptoms following face injuries.

## Introduction

Gastroparesis is objectively delayed gastric emptying in the absence of mechanical obstruction leading to non-specific symptoms of nausea, vomiting, bloating, and upper abdominal pain [1]. It is a motility disorder, diagnosed after the exclusion of obstruction using imaging or endoscopy [2]. The prevalence of gastroparesis is higher in patients with diabetes (40% in type 2 and 10–20% in type 1) with the predominant symptom being vomiting whereas, idiopathic gastroparesis commonly presents with upper abdominal pain as the primary complaint [3, 4].

Primary management includes the management of symptoms, ensuring adequate nutrition, and improving motility and glycemic control in patients with diabetes [5].

Nutrition can be maintained enterally if tolerated or parenterally. Motility treatment along with symptom management can be utilized with antiemetics or prokinetics (metoclopramide or a short-term erythromycin use). Other techniques include centrally acting antidepressants, venting gastrotomy, feeding jejunostomy, or intra-pyloric botulinum toxin [5].

The disease is associated with a significant patient burden with direct and indirect economic consequences [5]. Along with the increased morbidity and mortality, an increase in direct health care costs through hospitalizations, emergency room visits, and doctor visits necessitate efficient diagnosis and management of gastroparesis [6]. The purpose of this case report is to illustrate the diagnostic and therapeutic challenges encountered in managing gastroparesis, highlighting a notable observation regarding the potential correlation between parasympathetic surges and symptom remission following facial trauma.

## Case presentation

**Fig. 1:**
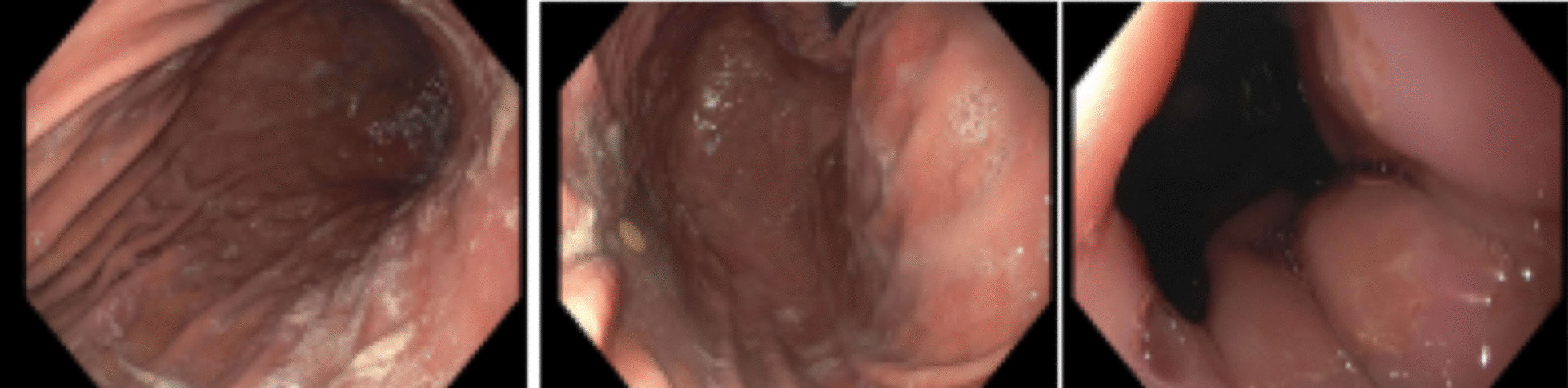
Upper endoscopy revealing normal gastric mucosa with no strictures

A 46-year-old Hispanic man with a past medical history of hypertension, T2D, and hyperlipidemia presented with a diabetic foot ulcer and suspected osteomyelitis, preceded by the resection of the fifth toe and an unsuccessful skin graft. An angiography revealed inadequate blood circulation in the left posterior tibial artery, which was subsequently enhanced with balloon angioplasty. Surgery planned for wound exploration was postponed due to exposed bone and macerated tissue, leading to admission for intravenous (IV) antibiotics for clinical osteomyelitis. Cultures grew methicillin resistance staph aureus (MRSA) and Enterococcus, resulting in antibiotic adjustments. However, the patient developed severe nausea and vomiting attributed to ampicillin/sulbactam. The gastroenterology team was consulted and recommended to continue antiemetic medication, encourage out of the bed to chair, give a trial of very small sips of clear liquids if tolerated, and get *Helicobacter pylori* (*H. pylori*) stool antigen which turned back positive and led to a change in antibiotic. Given the lack of improvement, the gastroenterology team was again consulted and recommended to get gastric emptying studies to rule out gastroparesis as the cause of emesis, to continue metoclopramide, and to start erythromycin. Gastric emptying studies revealed a gastric emptying time of 748 min. The normal values are 45–90 min. To rule out the possible effect of calcium channel blockade with amlodipine on gastric motility, amlodipine was held, and he started carvedilol; however, no improvement was observed. After completion of triple therapy for H. Pylori, he was still experiencing nausea and vomiting with minimal response to metoclopramide and erythromycin. An upper GI scope was done, which was unremarkable (Fig. 1); meanwhile, the patient required parenteral nutrition.

The patient was diagnosed with severe gastroparesis based on gastric emptying studies and upper GI scope. The gastroenterology team recommended either to get a percutaneous jejunostomy as an alternative method of feeding or to get a gastric pacemaker to help with the gastroparesis.

**Fig. 2:**
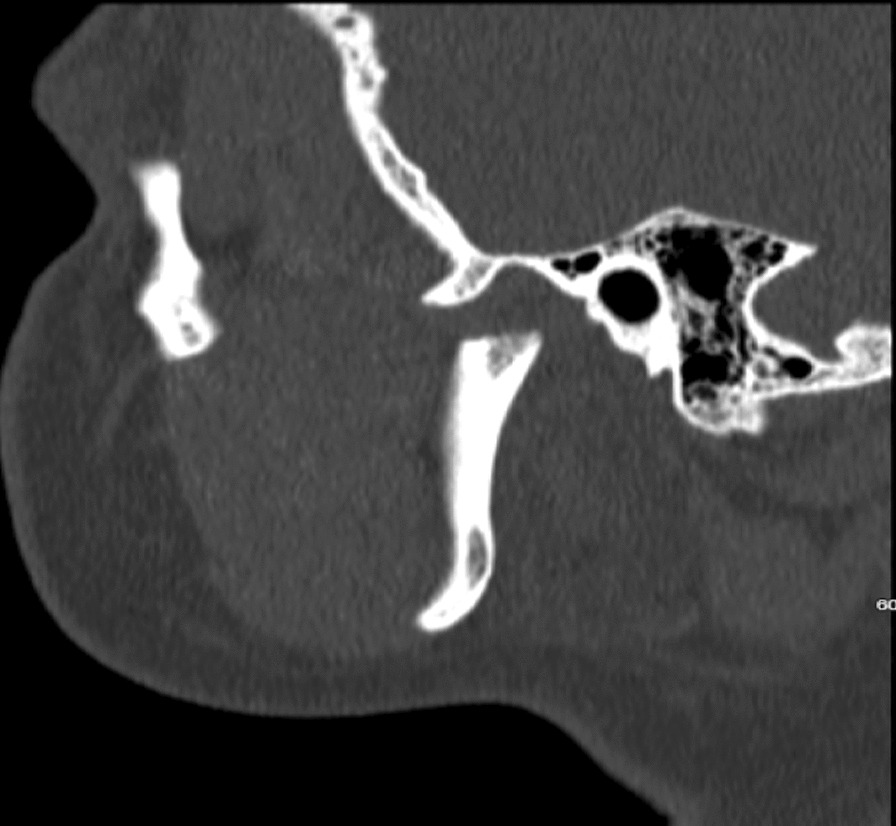
Computed tomography maxillofacial sagittal view demonstrating fracture of neck of right mandible

**Fig. 3:**
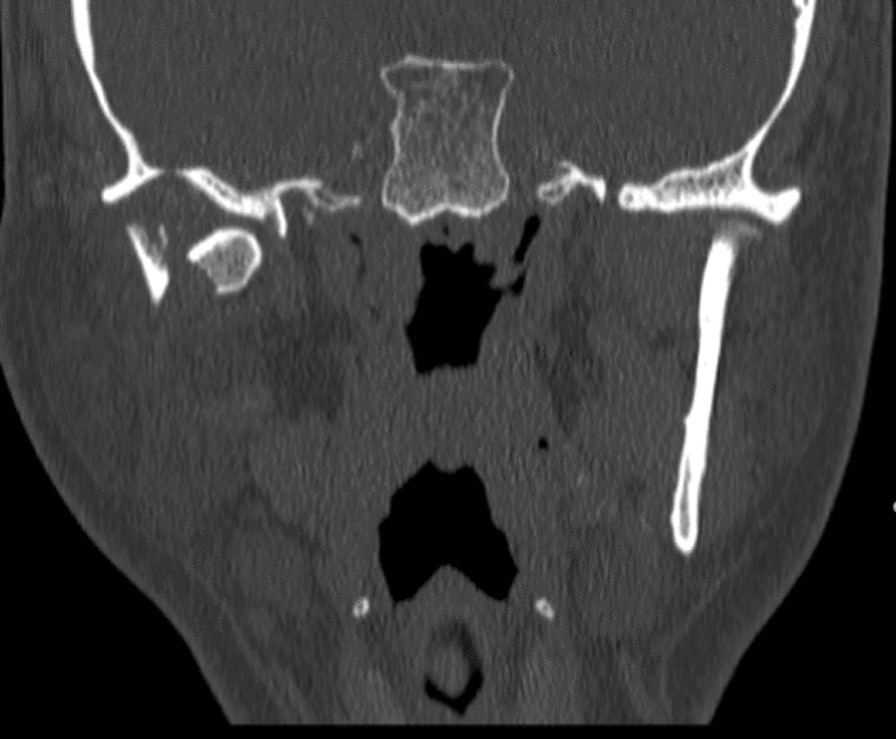
Computed tomography maxillofacial coronal view demonstrating fracture of neck of right mandible, the mandibular condyle is comminuted and displaced medially

**Fig. 4:**
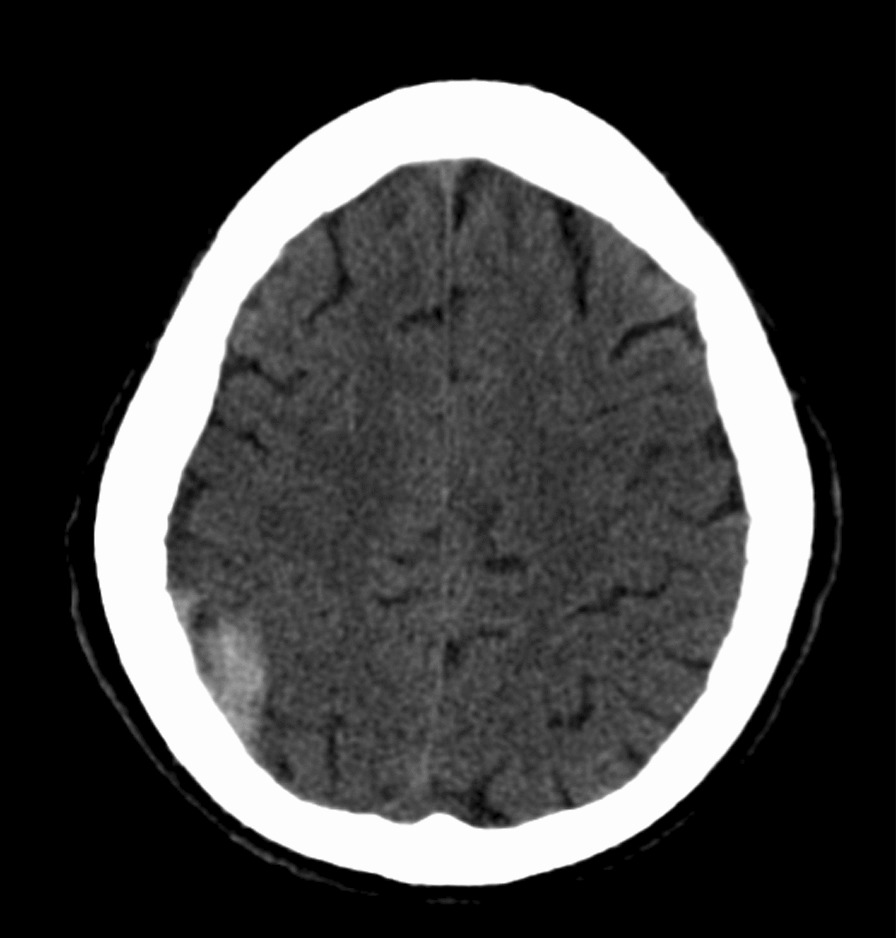
Computed tomography of the Head revealing a right posterior sub-acute epidural hematoma

**Fig. 5:**
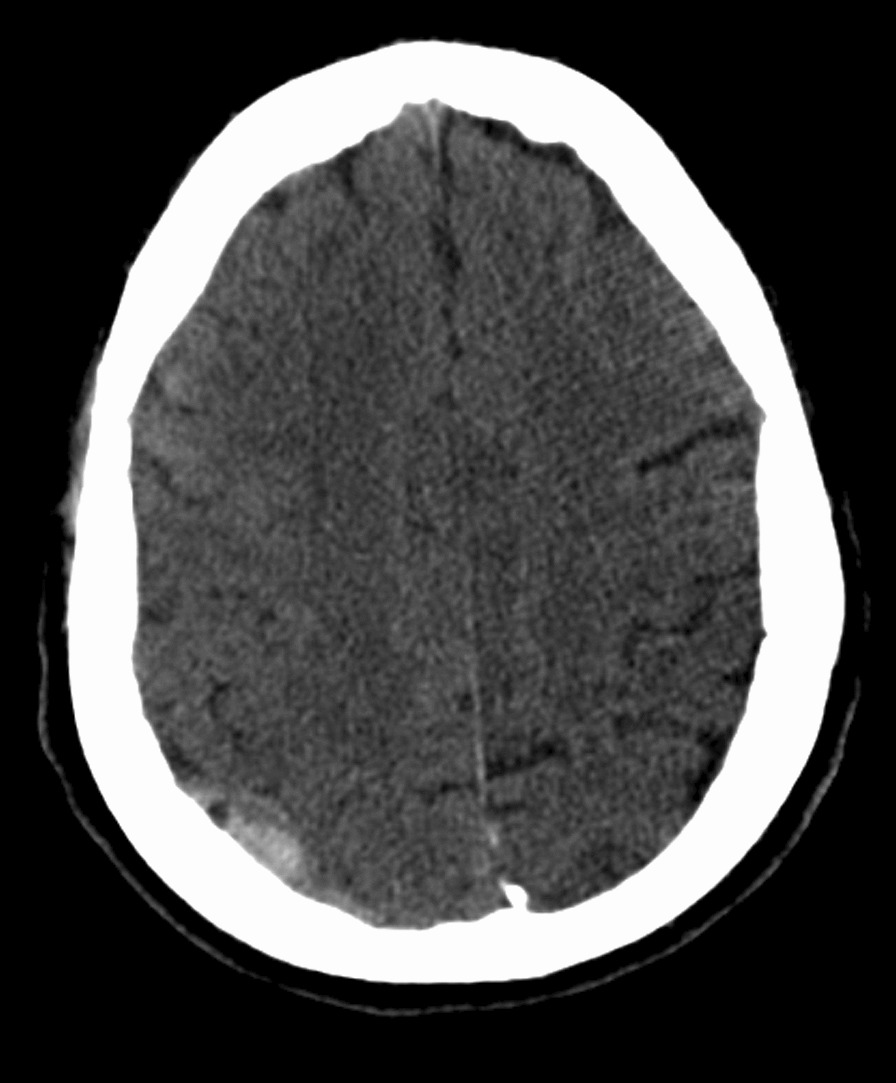
Computed tomography of the head demonstrating interval stable epidural hematoma

Unfortunately, the patient suffered a mechanical fall while going to the bathroom resulting in facial trauma. He received a laceration on the submandibular region and was complaining of right-sided facial pain. Computerized tomography (CT) maxillofacial was done which confirmed the patient has a fracture of the right mandibular (Figs. 2, 3). CT head showed the patient has a subacute epidural interval (Fig. 4); with interval CT head showed the bleeding was stable in size (Fig. 5).

Neurosurgery recommended no need for acute intervention, and to observe him for any seizures. The patient was started on levetiracetam for seizure prophylaxis and the patient was upgraded to intensive care unit (ICU) for further monitoring. Surprisingly, after the fall, the patient's nausea improved and emesis stopped. He was given a trial of oral liquids that he successfully completed. As the patient was able to tolerate an oral diet and was not complaining of nausea, metoclopramide and erythromycin were held.

The syncopal episode was likely due to orthostatic hypotension secondary to a suspected decrease in peripheral autonomic reserve, decrease in preload, and decrease in systemic vascular resistance from sepsis. The patient eventually completed 6 weeks of IV antibiotics with ampicillin/sulbactam and vancomycin. Since the fall the patient did not have any complaints of nausea or vomiting and apparently was able to tolerate the oral diet. Repeat gastric imaging or further intervention was deemed unnecessary as the patient subjectively was able to tolerate oral challenge. Eventually, the patient was discharged after the fixation of the mandibular fracture.

Improvement in gastroparesis was attributed to sudden parasympathetic surges resulting from facial trauma. The patient was eventually discharged to the community with outpatient follow-up with Ear, Nose and Throat (ENT), Gastroenterology, Cardiology, General Surgery, Plastic Surgery, and Neurosurgery. Currently, he is asymptomatic on a 1-year follow-up, denies any nausea, and is comfortable tolerating an oral diet.

## Discussion

Gastroparesis is a challenging condition that manifests as delayed gastric emptying in the absence of a mechanical gastric outlet obstruction [7]. The condition tends to be multifactorial and can significantly impact one’s quality of life. The true prevalence of gastroparesis is unknown; however, it is estimated that up to 4% of the general population presents with symptoms of gastroparesis [8]. Studies from tertiary academic centers have reported a high prevalence of gastroparesis in type 1 diabetes (T1D) (40%) and T2D (10–20%). Still, the data may be skewed and may not represent the community prevalence, which was estimated to be about 24.2 per 100,000 persons and an incidence of 6.3 per 100,000 person-years, both with a significant female predominance in a study based on Olmsted County in Minnesota by Jung et al. [9]. Our presented case of a 46-year-old male with gastroparesis highlights the clinicopathological profile of this patient population and the management hurdles that go together with this condition.

Though multifactorial, the most common subtypes of gastroparesis are idiopathic, diabetic, and postsurgical. Less common etiologies are neurodegenerative disorders like Parkinson’s disease, myopathy associated with conditions like scleroderma, amyloidosis and neoplastic syndrome [10]. In approximately one-third of the cases, gastroparesis is related to T1D or T2D, with uncommon etiologies like systemic lupus erythematosus, hypothyroidism, human immunodeficiency virus [11, 12], and inflammatory disorders of the GI tract [13] along with the abovementioned causes making up a third of patients. The remaining one-third is idiopathic, where the cause is not known. Our 46-year-old male developed gastroparesis, most likely in the setting of ongoing uncontrolled T2D.

Regardless of the etiology, gastroparesis symptom profiles usually include nausea, vomiting, early satiety, and postprandial fullness [14]. In a multicenter National Institute of Health gastroparesis registry for 416 patients, symptoms prompting evaluation for diabetic gastroparesis included vomiting, and for idiopathic gastroparesis, the predominant symptoms were abdominal pain with early satiety. All the patients on the registry had documented delayed gastric emptying in their medical records [4, 15]. According to Horowitz et al. repeated vomiting several hours after eating highly suggests gastroparesis [7]. A combination of symptoms and delayed gastric emptying time is required to diagnose gastroparesis, as the management of the condition is based on combined criteria**.** Jan Tack et al. discuss that although measurement of gastric emptying time is necessary for diagnostic labeling, it does not significantly explain the symptom pattern and prognostication of the disease [16]. The chief complaint in our case was refractory nausea and vomiting; gastroparesis was diagnosed considering the patient’s presentation and documented delayed gastric emptying time.

A delay in gastric emptying time can be established with scintigraphy, the gold standard, and several objective methods like stable isotope breath test, magnetic resonance imaging, and antropyloroduodenal manometry. GI endoscopy is warranted in every possible case of gastroparesis to rule out mechanical gastric outlet obstruction and any extraluminal pathology [17]. Management of gastroparesis is multifaceted, starting with treatment for upper GI symptomatology and electrolyte disturbances that ensue from the former. Dietary and lifestyle measures form the cornerstone of the management of gastroparesis. Dietary advice should focus on individual tolerances and difficulties with specific food groups. Patients are encouraged to eat smaller portions more frequently throughout the day and avoid late-night meals. They are also advised to reduce their intake of fatty food products and limit consumption of insoluble fibers as both food groups can further adversely affect the gastric emptying time. In severe cases of gastroparesis with refractory vomiting, enteral nutrition through a feeding jejunostomy tube [17] may be required for adequate nutritional support for the patient and to allow gastric recovery time [18]. Our presented case was given nutritional support with parenteral nutrition in the setting of intolerance to oral nutrition and electrolyte disturbances due to persistent vomiting. Planning for enteral nutrition options like feeding the jejunostomy tube coincided with the patient’s recovery of tolerance to oral intake. This resulted in cautious switching to partial parenteral nutrition along with oral intake of liquids and solids in supervised settings.

Among the prokinetic agents, metoclopramide, a dopamine D2-receptor antagonist, is the only food and drug administration (FDA) approved medication used to treat gastroparesis and, according to guidelines, should not be used for longer than 12 weeks [19] because of the risk of side effects like tardive dyskinesia [20]. Symptoms of diabetic gastroparesis improved with metoclopramide, which was assessed in 4 placebo-controlled trials. The drug’s long-term efficacy remains to be proven, as none of the four trials were conducted beyond four weeks [19]. Domperidone, a type II dopamine antagonist available through the FDA’s expanded access to investigational drugs program, has been used to treat gastroparesis; it is equally efficacious with a lower incidence of central side effects when compared to metoclopramide, as domperidone does not cross the blood–brain barrier [10]. A systematic review of 28 trials showed a symptomatic reduction in 64%, decreased hospitalization in 67%, and accelerated gastric emptying in 60% of patients with diabetic gastroparesis [15]. However, domperidone should be avoided in patients with QTc prolongation (> 470 ms in males, > 450 ms in females) [10]. Cisapride, a 5HT_4_ receptor antagonist, was efficacious in gastroparesis [21] but was withdrawn due to an increased risk of cardiac arrhythmias [22].

Synthetic cannabinoids, like dronabinol, have been used to treat nausea and vomiting of gastroparesis, but these agents can cause significant hyperemesis on withdrawal [23]. Neurokinin receptor-1 antagonist aprepitant [24] and transdermal scopolamine are also used to address vomiting associated with gastroparesis. Tricyclic antidepressants and selective serotonin reuptake inhibitors are among other medications used for gastroparesis [25, 26]. Macrolides like erythromycin, azithromycin, and clarithromycin are motilin receptor agonists with prokinetic properties [10]. Our 46-year-old patient initially received metoclopramide for managing nausea and vomiting; Trimethobenzamide, a dopamine D2 receptor antagonist that blocks impulses to the chemoreceptor zone, was tried during his hospital course. Erythromycin was another agent used in our patient to promote gastric emptying. Clinical studies have demonstrated effectiveness of levetiracetam which in prophylaxis against cyclic vomiting syndrome episodes, making it a potential treatment for a variety of pathologies; however, one of the known side effects of the medication itself is nausea and vomiting which make it less likely the reason for the improvement in the patients symptoms [27].

Several studies investigating new drugs for gastroparesis are underway. Relamorelin, a pentapeptide ghrelin receptor agonist, has shown preclinical evidence of having a robust prokinetic property [28], and in diabetic gastroparesis, the drug significantly accelerates gastric emptying of solids [29–31]. Trazpiroben, a dopamine D2/D3 receptor antagonist with minimal brain penetration, has been shown to improve postprandial symptoms in patients with diabetic gastroparesis [32]. In refractory cases, especially in the setting of weight loss and continued electrolyte disturbances, intrapyloric injection of botulinum toxin, implantable gastric electrical stimulation or gastric pacing, or even surgical partial gastrectomy are considered. Still, the efficacy of these options remains unproven, and they come with their share of serious complications. Gastric pacing involves the implantation of electrodes in the smooth muscle layer of the gastric wall by laparotomy or laparoscopy; the electrodes connected to a subcutaneously located device stimulate the smooth muscle layer electrically. Short-pulse stimulation improved nausea and vomiting, reduced hospitalizations, and overall quality of life in several open-label studies, and long-term stimulation improved gastric emptying in a single open-label study [18]. Our presented case did not require surgical management as he experienced symptom resolution with medical management.

## Conclusion

This scenario highlights the complex and challenging nature of managing gastroparesis, which often necessitates a mix of dietary modifications, pharmaceutical treatments, and occasionally surgical measures. Customized treatment programs are crucial for enhancing the quality of life for those suffering with gastroparesis. Based on this rare clinical entity, further exploration of novel therapeutic methods has the potential to significantly improve the management of this illness.

## Data Availability

All supplemental data are available with the corresponding Author upon request.
